# Autofluorescence as a Signal to Sort Developing Glandular Trichomes by Flow Cytometry

**DOI:** 10.3389/fpls.2016.00949

**Published:** 2016-06-28

**Authors:** Nick Bergau, Alexander Navarette Santos, Anja Henning, Gerd U. Balcke, Alain Tissier

**Affiliations:** ^1^Department of Cell and Metabolic Biology, Leibniz-Institute of Plant BiochemistryHalle, Germany; ^2^Center for Basic Medical Research, Martin-Luther University of Halle-WittenbergHalle, Germany

**Keywords:** fluorescence activated cell sorting, tomato trichomes, type VI trichomes, autofluorescence, *Solanum habrochaites*, development, density gradient

## Abstract

The industrial relevance of a number of metabolites produced in plant glandular trichomes (GTs) has spurred research on these specialized organs for a number of years. Most of the research, however, has focused on the elucidation of secondary metabolite pathways and comparatively little has been undertaken on the development and differentiation of GTs. One way to gain insight into these developmental processes is to generate stage-specific transcriptome and metabolome data. The difficulty for this resides in the isolation of early stages of development of the GTs. Here we describe a method for the separation and isolation of intact young and mature type VI trichomes from the wild tomato species *Solanum habrochaites.* The final and key step of the method uses cell sorting based on distinct autofluorescence signals of the young and mature trichomes. We demonstrate that sorting by flow cytometry allows recovering pure fractions of young and mature trichomes. Furthermore, we show that the sorted trichomes can be used for transcript and metabolite analyses. Because many plant tissues or cells have distinct autofluorescence components, the principles of this method can be generally applicable for the isolation of specific cell types without prior labeling.

## Introduction

Glandular trichomes (GTs) are hair-like structures that cover the surface of more than 30% of all land plants (Dell and McComb, 1979; Fahn, 2000). They have attracted special attention because the secondary metabolites they produce are used as natural pesticides (Weston et al., 1989; Snyder et al., 1993; Frelichowski and Juvik, 2001; Kennedy, 2003; Zhang et al., 2008), as fragrances and food additives (Schilmiller et al., 2008) and in some cases as drugs, such as the antimalarial sesquiterpene artemisinin (Weathers et al., 2011). In the last decades an increasing number of biosynthetic pathways of secondary metabolites produced in GT has been investigated, (Gang et al., 2001, 2002; Croteau et al., 2005; Covello, 2008; Sallaud et al., 2009, 2012) but so far little is known about the development and differentiation of the structures that produce these secretions, the GTs. The development of non-GTs in the model species *Arabidopsis thaliana* has been extensively investigated (Uhrig and Hulskamp, 2010), but whether this knowledge can be transposed to GTs is the object of debate (Serna and Martin, 2006). To investigate GT development, methods for the isolation of different developmental stages of GTs would be desirable. The elucidation of trichome specific metabolic pathways has been greatly facilitated by the production of transcriptome datasets from these specialized cells (Tissier, 2012). The first transcriptomes were generated by the Sanger sequencing method (Dai et al., 2010), e.g., in peppermint (*Mentha × piperita*) (Lange et al., 2000), but in recent years the emergence of next generation sequencing (NGS) technologies as an affordable and quasi-exhaustive means of investigating the transcriptome space resulted in an explosion of datasets, particularly for non-model species (Unamba et al., 2015). More generally, the sensitivity and depth of NGS also stimulated the exploration of single cell types, both at the transcriptomic or epi-genomic level (Kanter and Kalisky, 2015; Angermueller et al., 2016; Clark et al., 2016). Single-cell data, whether at the transcriptome, epi-genome, proteome, or metabolome level, offers great promise in the elucidation of cell type-specific processes and for a better understanding of cell differentiation and the evolution of cell states. In humans for example, single cell biology approaches are being applied to dissect the cell identity landscape of tumors (Zhang et al., 2016) or for the identification of disease biomarkers (Niu et al., 2016). Single cell biology also offers great potential to advance our understanding of development and physiology in plants. However, despite a lower number of cell types than in mammalians, the presence of cell walls and of multiple cell layers makes the isolation of single cell types from plants, particularly from deeply embedded tissues, a challenging task (Schmid et al., 2015). One solution to this is to dissociate cells from one another by lysing the cell walls, i.e., by protoplasting. By combining this with tissue or cell-type specific expression of GFP, the corresponding cells can be isolated by fluorescence activated cell sorting (FACS). This was first successfully applied for transcriptomics of specific root tissues using microarrays (Birnbaum et al., 2003; Birnbaum et al., 2005; Nawy et al., 2005), and has been further extended to proteome and metabolite analysis of the *Arabidopsis* root (Petricka et al., 2012; Moussaieff et al., 2013; Petersson et al., 2015). This method, however, requires the establishment of transgenic GFP reporter lines and the relatively lengthy preparation times for protoplasts are unsuitable for the analysis of central metabolites, whose concentration changes within seconds or minutes (Misra et al., 2014). Laser-assisted microdissection (LAM) does not require prior labeling of specific cell types, but it is time consuming and the quantities of material recovered are small (Schmid et al., 2015). In addition, due to the fixation and embedding steps it is unsuitable for metabolite analyses. For cell types which can be easily accessed or identified direct sampling of the content of live cells by micromanipulation has also been successfully reported and used for transcriptome or metabolome analyses. Examples include the epidermal bladder cells (EBCs) of *Mesembryanthemum crystallinum*, the epidermal cells and idioblasts of *Catharanthus roseus* or tomato GTs (Oh et al., 2015; Nakashima et al., 2016; Yamamoto et al., 2016). However, rare cell types, or stages of cell or tissue differentiation which are transient, present additional challenges, in particular the isolation of sufficient quantity of material to perform subsequent analyses. Without enrichment, isolation of RNA from a few cells is possible and can be used for RNA sequencing after an amplification step, but the results present a high level of noise and, even with the help of sophisticated computational methods, have to be interpreted with caution (Wen and Tang, 2015).

In our endeavor to investigate GT development, we have chosen the tomato genus as a model system because of its excellent genetic resources and the availability of a high quality complete genome sequence including from related species (Sato et al., 2012; Tissier, 2012; Lin et al., 2014). Different techniques are known for the isolation of GTs (Gershenzon et al., 1987; Schilmiller et al., 2010; Balcke et al., 2014) but with these methods either purity, quality, or the amount of the isolated material is limited. Additionally none of the described methods allows the separation of different developmental stages, which is the main objective of this report. Tomato species (*Solanum lycopersicum* and related wild species) have different types of GTs, of which type VI represent the largest, with a diameter of about 60 μm, and most abundant type. The metabolites they produce include monoterpenes (in *S. lycopersicum*), various sesquiterpenoids and fatty acid derivatives such as methylketones in *S. habrochaites* (Fridman et al., 2005; Besser et al., 2009). These compounds, particularly in the wild species, contribute to an increased resistance against insect pests. We recently carried out a detailed analysis of the developmental stages of type VI trichomes in *S. lycopersicum* LA4024 and *S. habrochaites* LA1777 (Bergau et al., 2015). Although the development of type VI trichomes in both species follows a similar sequence, differences are observed in the structure of the trichome between the two species. The trichome head is round with a large internal cavity in *S. habrochaites* whereas it has four clearly visible cells and a small internal cavity in *S. lycopersicum*. The metabolites accumulate in this intercellular internal cavity and contribute to the high metabolite content of *S. habrochaites*, and thereby to a better protection against herbivores. In both species, type VI trichomes have a predetermined breaking point between the glandular cells and the intermediate cell (Bergau et al., 2015) and can therefore be harvested quite easily without much contamination by the bead–beating method first described by Gershenzon et al. (1987). However, this method, applied to tomato trichomes, does not allow the separation of young and mature trichomes. We previously showed that the early stages of type VI development in both species are characterized by the transient presence of a distinct green autofluorescence signal in UV light (Bergau et al., 2015). The identity of the fluorescent compounds could not be determined but preliminary results suggested they are of flavonoid origin (Bergau et al., 2015). Because these early developmental stages represent a very small fraction of the total trichome population, the green autofluorescence could be used as a specific signal for enrichment of the young trichomes by FACS. However, to the best of our knowledge, no method for cell sorting of small multicellular organs like trichomes based on a native autofluorescence signal has been described to date. Here we describe the development of a method for the preparation of pure fractions of young and mature type VI trichomes from *S. habrochaites* using FACS based on the autofluorescence of trichomes. We show that from these fractions high quality total RNA can be isolated and that specific metabolites can be identified. The procedure is based on a combination of sieving, density gradient centrifugation and flow cytometry and allowed us to separate and collect several thousands of young and mature trichomes in a short time.

## Materials and Methods

### Plant Material and Growth Conditions

Seeds of the wild tomato accession LA1777 (*S. habrochaites*) were obtained from the Tomato Genetics Resource Center, UC Davis, USA (tgrc.ucdavis.edu/). Plants were grown in a greenhouse with temperatures between 20 and 25°C. The humidity was maintained between 55 and 75%. The light intensity varied between 5 to 25 klux depending on weather conditions. Plant were treated once per week with a fertilizer solution (0.1% Kamasol Brilliant Blau, Compo Expert GmbH, Germany). trichomes were harvested from apices, whose size was approximately 1 to 2 cm, from 3 to 6 months old plants.

### Trichome Harvest

Trichomes were harvested by a bead-beating method modified from Schilmiller et al. (2010). Apices of LA1777 plants were harvested in 250 ml glass bottles, with about 200 apices per bottle, containing ice–cold sorbitol buffer (200 mM sorbitol, 50 mM Tris-Cl, 20 mM sucrose, 10 mM KCl, 5 mM MgCl_2_, 5 mM succinic acid, 1 mM EGTA, 0.5 mM K_2_HPO_4_, and 0.015% Triton X-100). To detach trichomes, 15 g of 0.75–1 mm glass beads were added and the bottles were shaken by hand for 2 min. This was followed by sequential sieving through 150, 63, 45, and 25 μm steel sieves. For RNA extraction, the harvest of apices and the sieving was done in 70:30 ethanol:water instead of the sorbitol buffer. The trichome fraction on top of the 25 μm sieve containing young and mature type VI heads was collected with concentrated by centrifugation for 5 min at 750 *g* and used for further enrichment.

### Density Gradient Centrifugation

Pre-purification of type VI trichomes was done with a stepwise density gradient centrifugation modified from Sallets et al. (2014). A Percoll^®^ gradient of four layers, each containing 3 ml of a mixture of Percoll^®^/sorbitol-buffer or Percoll^®^/ethanol was prepared. The Percoll^®^-concentrations were 30, 45, 55, and 80% (v/v) for both gradients. The trichome fraction was resuspended in 2 mL of sorbitol-buffer or ethanol:water (70:30) and loaded on top of the gradient and centrifugation was done for 15 min at 10 000 *g* and 4°C (Beckmann-Coulter Avanti J-30I, Rotor JS24.15). After centrifugation, the layers between 45 and 55% Percoll^®^, enriched in young type VI heads, and between 55 and 80% Percoll^®^, enriched in mature trichomes, were collected. Finally, trichome fractions were washed with 5 volumes of sorbitol-buffer or ethanol:water (70:30), the supernatant was discarded and the trichomes were stored on ice until further separation by fluorescence-activated cell sorting (FACS).

### FACS

For RNA isolation, the ethanol in the trichome fraction was replaced by washing the samples with phosphate buffered saline, to prevent trichome aggregation, and resuspending the washed samples in RNAlater^®^ (Thermo Fisher Scientific). To avoid any aggregates in the samples which could disturb the sorting, the preparation was sieved through a 50 μm nylon filter. For flow cytometry analysis, a FACS cell sorter Aria II (BD Biosciences, Heidelberg, Germany) was used. Although the Aria II was equipped with four lasers (blue, UV, red and yellow-green), only the blue laser was used for sorting. The use of the other lasers did not improve the discrimination of the autofluorescence of the trichomes. The trichomes were sorted using the 100 μm nozzle and the threshold of the flow cytometer was set at the channel for the Alexa-488-fluorescence. A dot plot of the forward scatter area (FSC-A) versus the forward scatter height (FSC-H) allowed discriminating single from aggregated particles. Although the young trichomes are different in size in comparison to the mature trichomes the FSC parameter did not allow discrimination between these particles. An optimal discrimination between the young and mature trichomes could be achieved through combination of the dot-plots Alexa-fluor 488 vs. PerCP and FSC-A vs. SSC-A.

For RNA isolation, the sorted trichomes were collected in RNA/DNA LoBind tubes (Eppendorf) containing RNA isolation buffer. For metabolite analysis, the trichomes were collected in empty LoBind tubes. The exact number of collected trichomes was determined by the cell sorter.

### Microscopy

All microscopic analysis was carried out on an AxioImager Z1 microscope (Zeiss, Jena, Germany). The different fractions of enriched trichomes were placed on a glass slide and observed in bright field and fluorescence with excitation of 450–490 nm and emission 515 nm long path. The enrichment of trichomes in the different fractions was determined by counting the trichomes in 2.25 μl of each fraction using a Neubauer improved hemocytometer (Fein-Optik, Jena, Germany).

### RNA-Extraction

To isolate RNA, 10 000 young and mature trichomes each were collected in RNA/DNA LoBind microcentrifuge tubes (Eppendorf) containing 100 μl RNA-extraction buffer. RNA extraction was performed using a RNA NucleoSpin XS Kit (Macherey–Nagel) following the user’s guide with the variation that the carrier RNA supplied in the kit was replaced by 20 μg linear polyacrylamide (Sigma–Aldrich). RNA was eluted in 10 μl water in 1.5 ml LoBind microcentrifuge tubes and immediately frozen and stored at -80°C. RNA integrity was tested by analyzing approximately 1 ng total RNA with a Picochip (Bioanalyzer 2100, Agilent).

### Metabolite Extraction and LC-MS

For metabolite analysis, 20 000 young and mature trichomes were collected in 1.5 ml tubes. Secondary metabolites were extracted with 500 μl of methanol/water 3:1 by shaking for 10 min at room temperature. Afterward, samples were centrifuged for 3 min at 15.000 *g* and the supernatant was collected. Subsequently, a second extraction of the pellet was conducted with 500 μl of methanol/formic acid 3:1. The two extracts were diluted to 1.3 ml with methanol to give a final concentration of 40% before analysis by LC-MS.

Online Solid Phase Extraction-LC-MS (SPE-LC-MS) analysis was performed with a Symbiosis Pico UHPLC (SPARK Holland B.V., Holland) coupled to a QTrap 6500 mass analyzer (SCIEX, Toronto, CA, USA). For SPE enrichment of the metabolites, Resin GP cartridges (SPARK Holland) were used. Conditioning and equilibration of the cartridges were done with 1 ml acetonitrile, 2 ml acidified water (1% acetic acid) and 2 ml water. Afterward, 1 ml of the trichome extracts was loaded on the SPE cartridge using a flow of 500 μl/min water on the cartridge. After loading, the cartridge was washed with 100 μl water. Thereafter, the SPE cartridge was put in line with the analytical column (Nucleoshell RP18plus column 2.7 μm, 2 mm × 150 mm, Macherey–Nagel, Düren, Germany) and eluted by the LC gradient pump for 6 min with a flow of 50 μl/min of 70% acetonitrile (solvent B) and 30% 0.3 mM ammonium formate pH 6.2 (solvent A). Peak refocussing at the head of the analytical column was achieved by adding acidified water (pH 3) to the eluent flow by a high pressure dispenser (HPD) at a rate of 250 μl/min. After 6 min, the gradient pump was set to 95% solvent A in order to equilibrate the starting conditions of the following LC separation (2 min). During this time the HPD continued to supply acidified water at 250 μl/min. To minimize carry-over, each sample was loaded on a new SPE cartridge. The LC separation of the metabolites was done by reversed phase chromatography at a column temperature of 40°C using a gradient of 0.3 mM ammonium formate pH 6.2 (solvent A) and acetonitrile (solvent B). The run time of the gradient was set to 24 min with the following profile: 0–6 min isocratic 30% A and 50 μl/min flow; 6–6.1 min linear from 30 to 95% A with 50 μl/min flow; 6.1–8.0 min isocratic 95% A and 50 μl/min flow. After this SPE-elution/peak-focusing period the column flow was increased to 400 μl/min. The gradient program was as follows: 8.0–8.1 min isocratic 95% A linear from 50 μl/min to 400 μl/min; 8.1–19 min linear from 95 to 5% A; 19–21 min isocratic 5% A; 21–22 min linear from 95 to 30% A; 22–24 min isocratic 30% A. MS detection was done by ESI-MS in negative mode with the following parameters: curtain gas = 40, ionspray voltage = -4500 V, temperature = 450°C, gas1 = 60, gas2 = 70, dp = -70 V, ep = -10 V, cxp = -10 V. For the detection of the secondary metabolites by multiple reaction monitoring (MRM), the following MRMs were used: tetramethylmyricetin m/z = 373.1/358.1 with collision energy of -33 V, kaempferol-diglucose m/z = 609.1/284.0 with collision energy of -46 V. The dwell time was set to 17 ms, each. The identity of the compounds was elucidated by exact mass measured by LC-QTOF (QTOF 5600, SCIEX, Toronto, CA, USA) and the MS/MS fragmentation spectra. For this analysis, an extract of unsorted trichomes was used.

## Results

Because the development of trichomes is rapid and not synchronous, trichomes at early stages of development are rare, even in young apices. The extremely low frequency of young trichomes in crude preparations (see below) prevented the direct use of FACS and required a preliminary enrichment.

### Harvest of Type VI trichomes and Pre-enrichment in Young Trichomes for Metabolite Analysis

**FIGURE 1:**
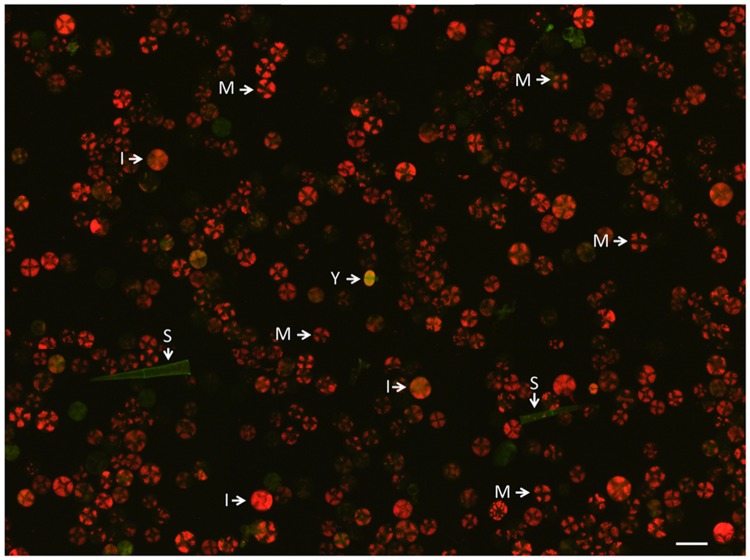
**Fraction of type VI trichomes after harvest in sorbitol-buffer by the bead-beating method before sieving and cell sorting.** The image was assembled from nine photographs and illustrates the low frequency of young trichomes (indicated by Y) at this stage of the preparation. The green structures are empty or possibly senescing trichomes. M, mature type VI trichomes; I, intermediate type VI trichomes; S, stalk, most likely from a type I or III trichome.

**FIGURE 2:**
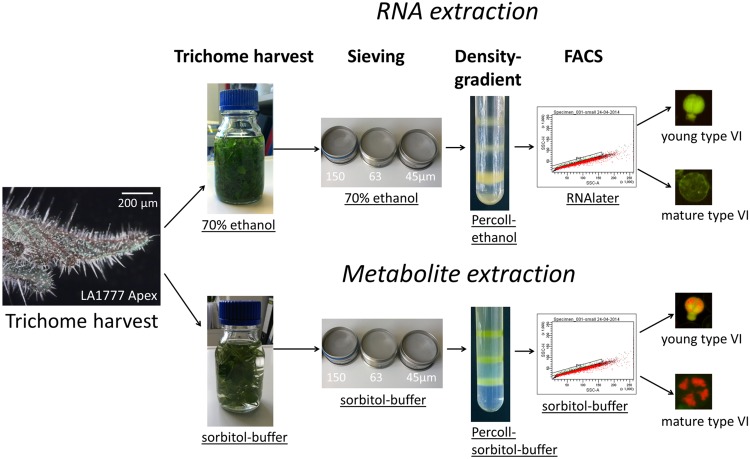
**Workflow for the separation of young and mature type VI heads for RNA and metabolite extraction**.

**FIGURE 3:**
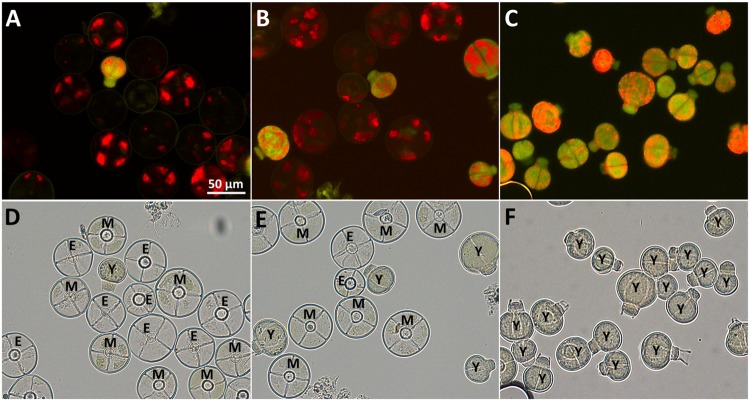
**Purification of type VI trichomes harvested in sorbitol-buffer**. Autofluorescence **(A–C)** and bright field microscopy **(D–F)** of fractions enriched in young type VI trichomes after sieving **(A,D)**, density gradient **(B,E)** and flow cytometry sorting **(C,F)** showing young (Y), intermediate (I), mature (M), and empty (E) type VI heads. Empty heads are likely due to damage done to the heads during the harvesting procedure.

Heads of type VI GTs were harvested from the wild tomato species *S. habrochaites* LA1777 according to Schilmiller et al. (2010) with minor modifications. During harvest, large amounts of sticky secondary metabolites are released and lead to aggregation of trichomes heads and stalks. This problem could be solved by adding 0.015% (volume per volume) of Triton X-100 to the isolation buffer. To increase the frequency of type VI trichomes at the early developmental stages, the harvest was done with apices of *S. habrochaites* LA1777. In first attempts, after harvest and sieving through a 150 μm mesh, samples were filtered through a 25 μm mesh on which the trichomes were retained. This fraction contained less than 0.7% (**Table 1**) of young type VI trichomes (**Figure 1**). This was still too low for enrichment by flow cytometry due to extended times required for sorting and resulted in a poor yield in the final fractions. To improve the pre-enrichment of young type VI trichomes, intermediate sieving steps and a density gradient centrifugation were added (see the workflow in **Figure 2**). Thus, the majority of mature trichomes, stalks, and leaf debris were removed by sequentially filtering the trichomes through 150, 63, and 45 μm steel sieves. With these improvements, the trichome fraction on the 25 μm sieve consisted of 2.3% of young type VI trichomes (**Figures 3A,D**; **Table 1**). Further enrichment of young type VI trichomes was achieved by density gradient centrifugation with a stepwise Percoll^®^-sorbitol buffer gradient as described previously for tobacco trichomes (Sallets et al., 2014). Young type VI trichomes have a lower density than mature ones (Supplementary Figure S1), allowing the collection of an enriched fraction with about 22.6% of young type VI trichomes (**Figures 3B,E**; **Table 1**). This fraction was used for the final separation by flow cytometry.

**Table 1:** Number of trichomes and percentages of the type VI trichome development stages during purification with the sorbitol-buffer method.

Step \ Fraction	Number of trichomes	Young type VI trichomes	Intermediate type VI trichomes	Mature type VI trichomes	Type VII trichomes	Stalks
After harvest	10 725 925 ± 637 252	0.68%	0.82%	96.23%	0.48%	1.79%
After sieving	2 629 629 ± 114 036	2.27%	1.14%	92.65%	1.70%	2.24%
After density gradient	225 185 ± 45 776	22.62%	0.29%	74.63%	2.18%	0.28%
After FACS	3 120 ± 891	>99%	<1%	<1%	NP	NP

*Trichomes were harvested from 800 apices of 80 plants. Values are average of three replicates. NP, not present.*

### Harvest and Pre-purification of Type VI Glandular Trichomes for RNA Extraction

In the method described above, the procedure of sieving and centrifugation without RNA stabilizing agents leads to loss of RNA integrity (Supplementary Figure S2A). Thus, for RNA isolation, the same procedure was applied but the trichomes were harvested and pre-purified in 70% ethanol and sorted in a solution containing RNAlater^®^ (**Figure 2**). When replacing the sorbitol buffer by 70% ethanol, the harvest and sieving of the type VI heads still yielded a type VI trichome fraction with a comparable enrichment for young trichomes, although the yield of the trichome fraction was slightly decreased (Supplementary Figures S3A,D and **Table 2**). To prevent RNA degradation throughout the procedure, the density gradient centrifugation was also done in a mixture of ethanol and Percoll^®^. This resulted in a qualitatively similar separation of the different developmental stages of the type VI trichomes in comparison to the Percoll^®^-sorbitol buffer gradient. However, the fraction containing the young type VI trichomes was significantly contaminated with type VII trichomes (Supplementary Figures S3B,E; **Table 2**). After the gradient centrifugation, the RNA extracted from the trichome fraction was of high quality (Supplementary Figure S2B). For the subsequent cell sorting step, the ethanol-Percoll^®^ mixture was exchanged with an aqueous RNAlater^®^ stabilization solution.

**Table 2:** Number of trichomes and percentages of the type VI trichome development stages during purification with the 70% ethanol method.

Step \ Fraction	Number of trichomes	Young type VI trichomes	Intermediate type VI trichomes	Mature type VI trichomes	Type VII trichomes	Stalks
After harvest	7 577 778 ± 1 424 824	1.67%	3.42%	90.18%	1.40%	3.33%
After sieving	2 925 926 ± 768 623	3.36%	1.78%	88.07%	2.72%	4.07%
After density gradient	114 444 ± 26 200	39.74%	2.43%	38.21%	14.02%	5.60%
After FACS	6 210 ± 1 368	>99%	<1%	<1%	<1%	NP

*Trichomes were harvested from 800 apices of 80 plants. Values are average of three replicates. NP, not present*.

### Sorting of Type VI Trichome Heads Using FACS

**FIGURE 4:**
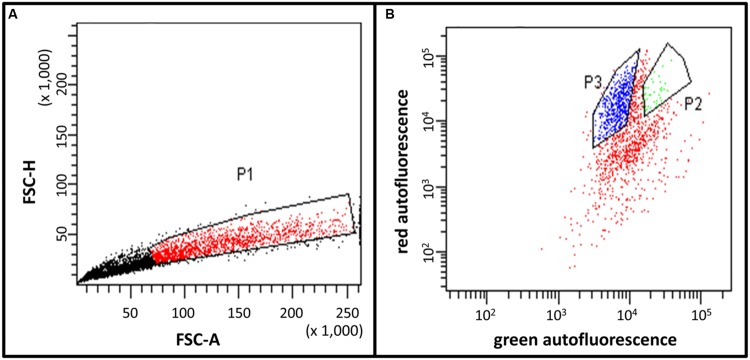
**Fluorescence activated cell sorting (FACS) histograms of the trichome fraction harvested in sorbitol-buffer. (A)** Preselection of young type VI trichomes in region P1 by the height of the forward scatter (FSC-H) and the area of the forward scatter (FSC-A). This allows the discrimination of single trichome heads and aggregates. The P1 region was selected for further separation based on differential fluorescence as shown in **(B)**. **(B)** Separation of young type VI trichomes (region P2) and mature type VI trichomes (region P3) based on different ratios between red (PerCP channel) and green (AlexaFluor 488 channel) fluorescences. **(A,B)** The units are arbitrary units provided by the instrument.

The final separation of type VI trichomes in pure fractions of young and mature type VI glandular heads was achieved by FACS. The signal of the FACS instrument was triggered by the fluorescence emitted with the blue laser on the AlexaFluor 488 channel. This allowed the best discrimination of the trichomes from the non-fluorescent particles in the sample. Additionally, dirt particles and aggregates were discriminated by defining regions on the FSC-A versus the FSC-H and FSC-A versus the side scatter area (SAC-A) dot-plots (**Figure 4A**). The autofluorescence of the selected trichomes was then analyzed (**Figure 4B**). This revealed that trichomes have two main autofluorescence signals, a red fluorescence due to the presence of chlorophyll in chloroplasts as detected via the PerCP channel, and a green fluorescence due to the autofluorescence of cell wall structures and the cytosolic signal of the young type VI trichomes as detected in the Alexa-fluor 488 channel. The fluorescence intensities varied strongly between the trichome preparations harvested in sorbitol-buffer or in ethanol. The preparation with ethanol lead to extraction of large parts of the chlorophyll thereby decreasing the red fluorescence detected in the PerCP channel. However, the green fluorescence and some of the red fluorescence persisted and were used for the separation. After preselecting region P1, which allowed eliminating aggregates of trichome heads (see Materials and Methods), single particles, young and mature type VI glands segregated in two distinct populations with different intensities in the Alexa-fluor 488 as well as in the PerCP fluorescence channels (**Figure 4B**). Separation of young type VI trichomes from type VII trichomes was more difficult but could be achieved by slight differences in the Alexa-fluor 488 and PerCP fluorescences. Finally, a trichome fraction containing 99% of young type VI trichomes could be collected (**Figures 3C,F**; Supplementary Figures S3C,F).

To obtain a comparable fraction containing exclusively mature trichomes, a parallel separation procedure was carried out with the fraction of the mature trichomes isolated from the density gradient (fraction b, see Supplementary Figure S1). Also here a fraction of almost 100% purity could be obtained from FACS. The resulting fractions of young and mature trichomes from the sorbitol and ethanol preparation methods were collected and used for metabolite and transcript analysis, respectively.

### Analysis of Metabolites and Transcripts of Young and Mature Trichomes

We next sought to demonstrate the applicability of this trichome separation technique to RNA and metabolite analysis.

**FIGURE 5:**
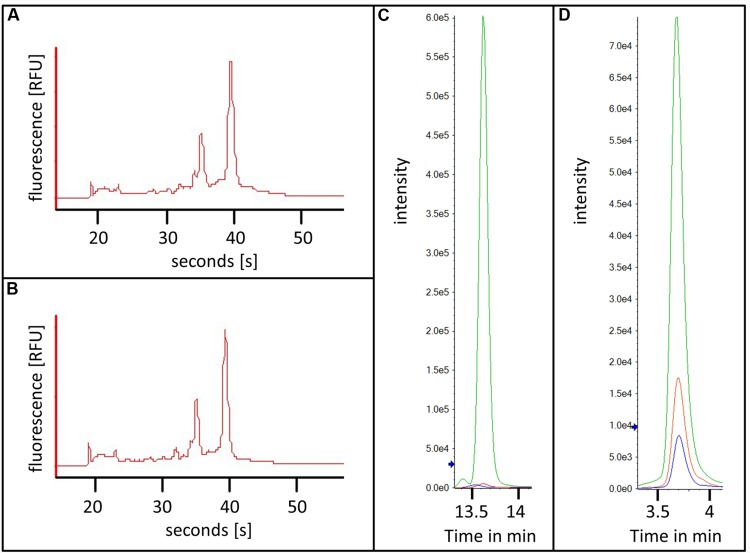
**RNA and metabolites extracted from flow cytometry sorted trichomes. (A,B)** Capillary electrophoresis chromatograms of RNAs from flow cytometry sorted young **(A)** and mature **(B)** trichomes; **(C,D)** Extracted ion chromatograms of kaempferol-diglucose **(C)** and tetra-methylmyricetin **(D)** from young trichomes in green and mature trichomes in red, water control in blue.

For RNA extraction, 10 000 young and mature trichomes were collected in RNA isolation buffer with the procedure described above. 17.8 and 8.7 ng of RNA could be recovered from young and mature trichomes, respectively. After the extraction of total RNA, the quality of the RNA was estimated by capillary electrophoresis (**Figures 5A,B**). The chromatograms displayed clear peaks for the ribosomal RNAs and almost no degradation products. This showed that the RNA of the trichomes stayed intact throughout the whole process of trichome harvest and sorting. Using this material, further RNA analysis such as RNA-sequencing or microarray hybridization could be done.

Next, we sought to demonstrate that the isolated trichome fractions can also be used for studying the metabolism of developing type VI glands. For this, acidic extracts of 20 000 young and mature trichomes were prepared and the levels of a number of flavonoid conjugates were analyzed (see list of MRM transitions used in **Supplementary Table S1**). Due to the relatively small number of cells that were used for this experiment the amount of the analyzed metabolites was expected to be very low and therefore difficult to detect. This issue was overcome by injecting the whole extracts with an online solid phase extraction system before the separation and by analysis of the metabolites by reverse-phase chromatography coupled to a QTRAP mass spectrometer (see Materials and Methods). The combination of the large volume injection with a very sensitive mass analyzer resulted in high signals and sharp peaks of the analyzed flavonoid conjugates. Two examples are shown in **Figures 5C,D** namely for kaempferol-diglucoside and for tetramethyl-myricetin. The mass spectra of these two compounds are shown in Supplementary Figure S4. Both secondary metabolites were measured in the mature and the young trichome sample. Interestingly, the levels of both metabolites were much higher in the young trichomes in comparison to the mature ones. In addition to demonstrating the adequacy of the preparation method for the analysis of secondary metabolites from the sorted trichome samples, these results also suggested that the flavonoid metabolism in young type VI trichomes is higher than in matured trichomes. This correlates well with the recently published data indicating the presence of a flavonoid like substance in the early stages of trichome development (Bergau et al., 2015).

## Discussion

In this study, we demonstrate the applicability of flow cytometry sorting to multicellular plant structures without digesting the cell wall and without the need of establishing a fluorescent reporter line. Flow cytometry has already been used to sort specific plant cell types but by the use of fluorescent markers which require the generation of transgenic lines expressing the marker under the control of a specific promoter and separating the cells digesting the cell walls (Birnbaum et al., 2003). There is also a report about using autofluorescence for flow cytometry of neutrophils (Dorward et al., 2013).

The main reason for developing this trichome isolation method was to prepare fractions enriched in the early stages of GT development for transcriptomics. These stages are poorly represented (i.e., less than 0.7%) using currently available trichome preparation methods and therefore particularly difficult to isolate. As shown in **Tables 1 and 2**; **Figure 3F**, the method allowed the preparation of highly enriched fractions of developing type VI trichomes (about 99%). To achieve this level of enrichment, we used a succession of harvest and fractionation techniques which, to the best of our knowledge, had not been combined together so far. This started with the bead-beater method, which is now well established for the harvest of GTs, followed by a succession of sieves of decreasing pore sizes and a density gradient centrifugation step, which had been previously applied for the preparation of pure fractions of tobacco trichomes (Sallets et al., 2014). After these steps, the fraction contained a sufficient proportion of young type VI trichomes to be processed by FACS based on the native fluorescence signals.

Our main aim was to develop a method to get RNA of sufficient quality and quantity for transcriptome analysis of young trichomes. One difficulty encountered here was that the sorbitol buffer traditionally used for the harvest of GTs (Schilmiller et al., 2010; McDowell et al., 2011) resulted in RNA samples of insufficient quality for downstream applications due to extensive degradation. This is likely due to the relatively long times (5 to 6 h in total from harvest to FACS) required to prepare the fractions. This problem was solved by replacing the sorbitol buffer with a 70% ethanol solution, which effectively inactivates RNAses (Clément-Ziza et al., 2008; Dobritzsch et al., 2015). With this ethanol solution, chlorophyll and probably many other metabolites, were partially extracted. Because the chlorophyll fluorescence was used in the FACS, the sorting parameters had to be adjusted accordingly. Interestingly, the green fluorescence signal characteristic of the young trichomes was unaltered, indicating that although the compound is suggested to be a flavonoid (Bergau et al., 2015), it does not seem to be soluble, possibly because it is bound to a polymeric structure. Despite its inadequacy for RNA extraction, the sorbitol buffer method could be used for metabolite extraction of trichome fractions. However, it should be noted that, because of the relatively lengthy preparation procedure, this method is unlikely to be useful for the analysis of primary metabolites, whose levels are known to alter very rapidly upon dramatically changing conditions (Tredwell et al., 2011).

The amount of RNA that was extracted from young trichomes (10–20 ng range) would be sufficient to perform high quality RNA-sequencing with amplification. A recent survey shows that although RNA-seq data can be obtained from sub-nanogram quantities of RNA, the number of detected genes and the significantly lower correlation of gene expression values between replicates suggest that it is preferable to start with larger quantities of RNA, such as 5 ng (Shanker et al., 2015). Single-cell biology, and particularly transcriptomics, is generally perceived as a powerful approach to a better understanding of a variety of processes, particularly development and cellular differentiation. A distinction has to be made, however, between truly single-cell transcriptomics, where RNA is extracted from individual cells, and single-cell type transcriptomics, where RNA is extracted from a population of one cell type, usually from several hundred to several thousand cells. In these cases, the low amounts of RNA that are recovered require extensive amplification before sequencing, resulting in noisy data which require cautious interpretation, particular for genes that are expressed at low levels (Wen and Tang, 2015). Even though computational methods are now available to improve the analysis of these datasets, it is, in our opinion, preferable to get larger quantities of RNAs for single-cell type populations. With the method described here, it is possible to harvest within hours a large number (several tens of thousands) of GTs that are in the early stages of development. Even though this population is not perfectly homogeneous – two celled and four-celled GTs are present (**Figure 3F**) – it still provides an unprecedented view into the early stages of GT development an differentiation, which until now has not been investigated by global approaches due to the transient nature of these stages and thereby their rarity.

Direct sampling using micro-manipulation is an alternative to the method we describe here. This was successfully done for example for the transcriptome analysis of EBC of the ice plant (*M. crystallinum*), where the content of 3000 EBCs was collected using a 27G needle (Oh et al., 2015). This was possible because the whole epidermis of ice plant leaves are covered with EBCs, providing a large number of cells of the same type, and because these cells are very large. 27G Needles have an external diameter of 0.41 mm which would make them useless for the collecting of developing trichome content, whose size does not exceed 50 μM. However, recent publications describe the use of capillary probes to collect the content of single plant cells. This was done for the MS analysis of single cells of *C. roseus* and of different trichome types of tomato (*S. lycopersicum*) (Nakashima et al., 2016; Yamamoto et al., 2016). So far these sampling techniques have only been used for metabolite analysis and it remains to be seen whether they can also be applied for RNA extraction. Furthermore, these samplings have been carried out on cell types that are abundant (mature GTs) and easy to identify (fluorescence of the idioblasts and latificers of *C. roseus*). Collecting several thousands of early stage type VI trichomes required the processing of hundreds of tomato apices, a procedure which can be carried out within a day. Whether processing such large numbers of apices within this time frame using the micro-capillary probes is feasible, is questionable. Therefore, despite its limitations, the method described here is well suited for the collection of large numbers of a rare cell type.

One issue is the relevance of the method described here for other plant species and plant cell types. Plant cells produce a variety of autofluorescent compounds, including chlorophyll but also a large number of secondary metabolites as well as cell wall components (e.g., in lignin) (Roshchina, 2012; Talamond et al., 2015). Secondary metabolites frequently occur in specialized secretory cell types, such as GTs as used here, but also cells that are deeply embedded in the plant tissue. Examples are the idioblasts and laticifers of *C. roseus* mentioned previously (Yamamoto et al., 2016), but also the avenacin producing cells in oat roots (Turner, 1960). Upon pathogen infection, plant cells can also be elicited to produce defense compounds that are fluorescent (Roshchina, 2012). There are therefore many cases where endogenous autofluorescent compounds could be used to isolate specific cell types, particularly cells that are involved in the production of specialized metabolites. Unlike for trichomes, isolation of cells that are deeply embedded in plant tissues would require protoplasting, but this type of approach has been successfully applied for the isolation of specific cell types using fluorescent reporter lines (Moussaieff et al., 2013) and there is no reason why it could not be applied to endogenous autofluorescence signals. In addition, FACS is a powerful cell selection tool where multiple fluorescence signals can be monitored simultaneously, allowing refined sorting based for example on the ratio of different fluorescent signals or the parallel sorting of distinct cell types.

In conclusion, beyond providing the material for deeper insights into the developmental processes of GTs, the method described here, with the necessary adaptations, should prove useful for the study of specific plant cell types.

## Author Contributions

AT conceived the project. NB and AT designed the research. NB, ANS, HN, and GB performed all experiments. NB and AT wrote the manuscript.

## Conflict of Interest Statement

The authors declare that the research was conducted in the absence of any commercial or financial relationships that could be construed as a potential conflict of interest.

## Supplementary material

TABLE S1 **MRM (multiple reaction monitoring) transitions for the 37 analyzed flavonoid-conjugates indicating the selected masses of the precursor ion (Q1) and the fragment ion (Q3) as well as the collision energy used for the fragmentation**.
