# Supplementary material for: Autofluorescence as a Signal to Sort Developing Glandular Trichomes by Flow Cytometry
Source: Front Plant Sci. 2016 Jun 28;7:949. doi: 10.3389/fpls.2016.00949 (PMC4923063; doi:10.3389/fpls.2016.00949)

Supplementary material for the article by Bergau et al., "Autofluorescence as a signal to sort developing glandular trichomes by flow cytometry"

Supplemental Figure 1: Percoll®-density-gradient of type VI glandular trichomes harvested in sorbitol-buffer.

A: photograph of the Percoll®-density-gradient after centrifugation. B: Fraction B containing mature type VI trichomes (M) and type VI trichomes of an intermediate stage (I), C: Fraction C containing young (Y) and mature type VI trichomes (M). D: Fraction D containing mature type VI heads (M). The scale is the same for all microscopy images (B-D).

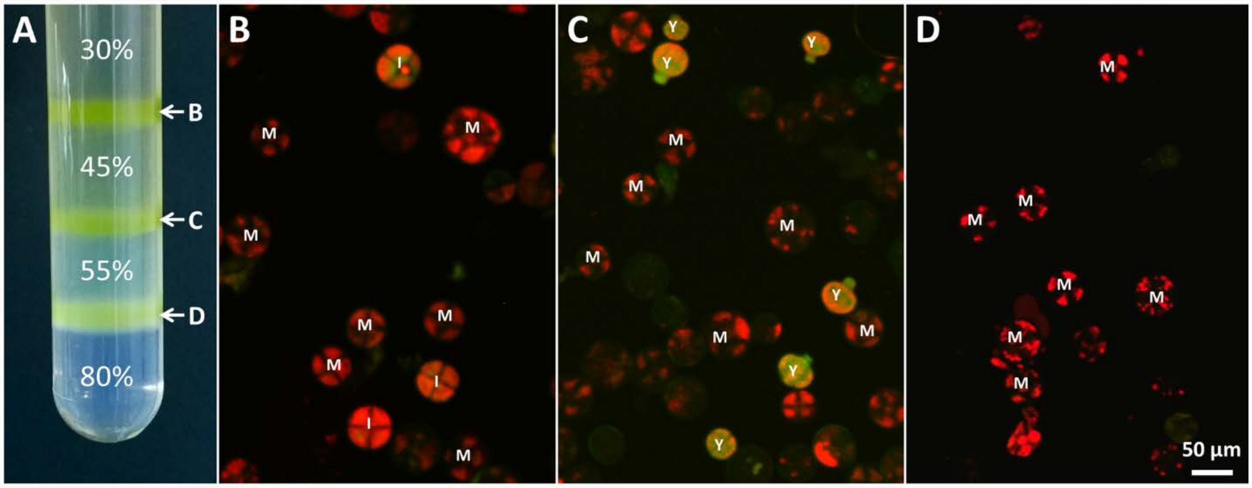

Supplemental Figure 2: RNA profiles of fractions enriched in type VI trichomes. A: after harvesting and sorting without any stabilizing agents. B: after harvesting and density gradient centrifugation in 70% ethanol. The profile in (A) shows extensive degradation, whereas in (B) clear peaks for ribosomal RNA can be seen and no significant degradation can be observed.

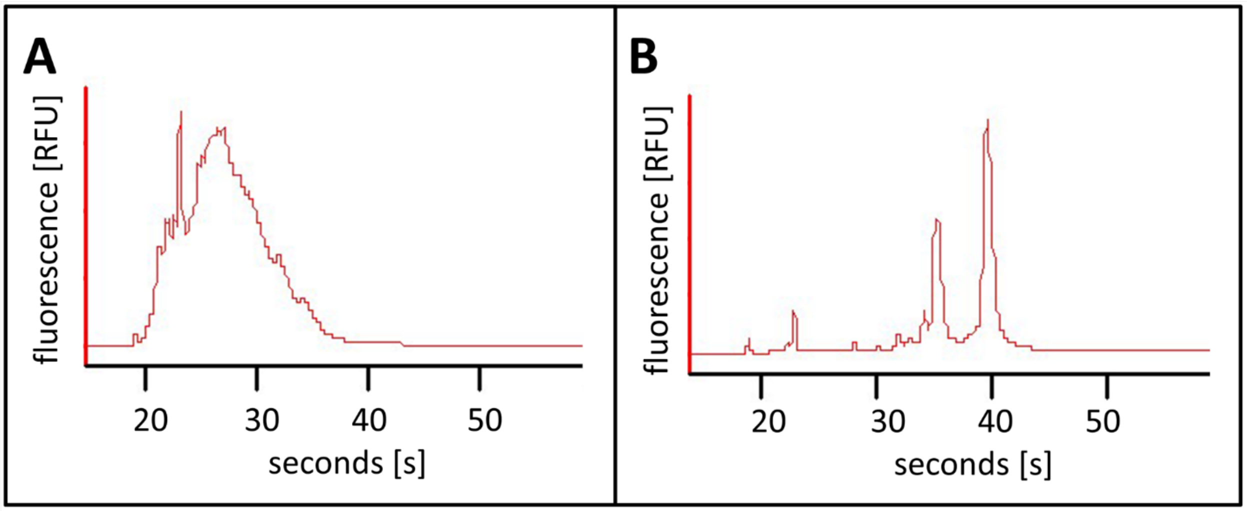

Supplemental Figure 3: Purification of type VI trichomes harvested in 70% ethanol. Autofluorescence (A-C) and bright field microscopy (D-E) of purified young type VI trichome fraction after harvest (A, D), density gradient (B, E) and flow cytometry sorting (C, F) showing young (Y) and mature (M) type VI trichomes, type VII trichomes (VII) and cell debris (D). The scale is the same for all images.

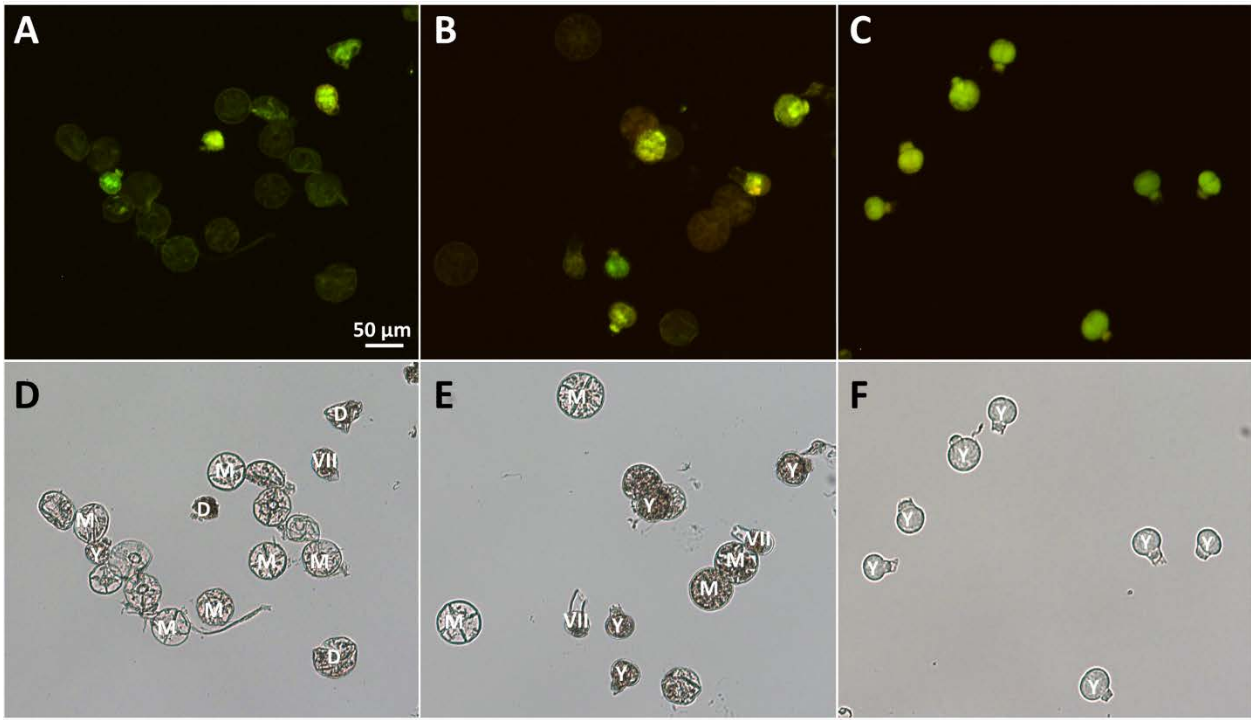

Supplemental Figure 4: Mass spectra of kaempferol-diglucose and tetra-methylmyricetin.

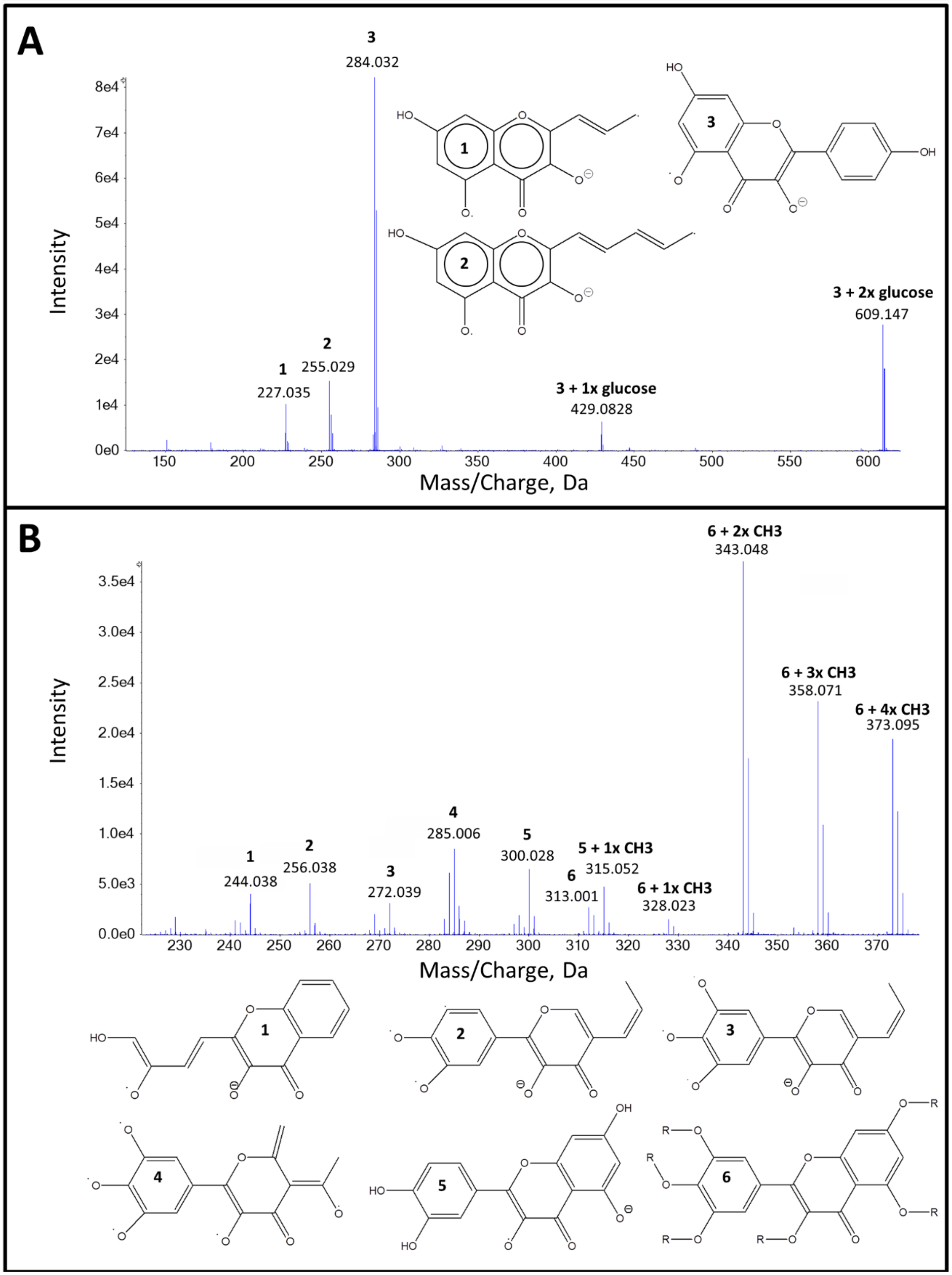

Supplement: Supplementary file 2 [file Data_Sheet_1.PDF]
